# Unusually Late Onset of Creutzfeldt-Jakob Disease Following COVID-19 Infection in India: A Case Report

**DOI:** 10.7759/cureus.63702

**Published:** 2024-07-02

**Authors:** Afreen Begum, Madhava S Boppana, Neha Sivani Rajavasireddy, Nikhila Tummala, María Belén Solís Mayorga

**Affiliations:** 1 Medicine, Employee State Insurance Corporation (ESIC) Medical College and Hospital, Hyderabad, IND; 2 Medicine, Guntur Medical College, Guntur, IND; 3 Medicine, Government Medical College, Vijaywada, Vijaywada, IND; 4 Neurology, Larkin Community Hospital, Miami, USA

**Keywords:** small vessel ischemia, hypoglycemic encephalopathy, creutzfeldt-jakob disease, encephalitis, diffusion-weighted mri

## Abstract

Numerous studies have demonstrated the rise in neurological and psychiatric issues linked to post-COVID-19 infections. The most prevalent symptoms include encephalopathy, seizures, depression, anxiety, and ischemic or hemorrhagic stroke. The occurrence of Creutzfeldt-Jakob disease (CJD) after COVID-19 was unusual, but recent studies have shown a connection between COVID-19 and prion disease. Most cases of CJD present within weeks or a few months after the onset of COVID-19. The late onset of Creutzfeldt-Jakob disease following the COVID-19 infection raises questions about the potential pathophysiological mechanisms underlying this association. Although the exact link remains elusive, this case adds to the growing body of evidence suggesting a possible relationship between COVID-19 and neurodegenerative diseases. Further research is warranted to elucidate the underlying mechanisms and optimize management strategies for post-COVID-19 neurological complications. We present to you an 83-year-old man with a history of COVID-19 infection who presents with memory impairment, mood instability, and declining cognitive function. Despite initial improvement, his condition rapidly deteriorated, ultimately leading to a diagnosis of probable Creutzfeldt-Jakob disease.

## Introduction

A rapidly advancing neurodegenerative condition known as Creutzfeldt-Jakob disease (CJD) is caused by an erroneous conformational change in the prion protein, which eventually spreads and builds up in the brain [1]. There are four types of CJD: iatrogenic, genetic, variant, and sporadic. With a mean survival of four to eight months, sporadic CJD affects 85% of cases. A mutation in the autosomal dominant PRNP gene, which codes for the prion protein, causes genetic CJD, which affects 10-15% of cases. In contrast to iatrogenic CJD, which can result from contamination during brain surgery, corneal transplants, or dura mater grafts, variant CJD is brought on by eating contaminated beef. The predominant subtype of CJD is still sporadic [2]. Patients between the ages of 55 and 75 typically develop CJD, and 90% of cases end in death after a year of illness [3]. The primary cause of coronavirus disease 2019 (COVID-19) is the SARS-CoV-2 coronavirus, whose high contagiousness resulted in a global pandemic that is still going strong today. In COVID-19 cases, a wide range of neurological complications have been noted, the most common of which are hyposmia, cerebrovascular diseases, headaches, cognitive deficits, and Guillain-Barré syndrome [4].

The complex and highly interactive disease mechanisms that characterize “long COVID,” PrD, neurodegeneration, and SARS-CoV-2 neurobiology are defined by the discovery of considerably overlapping pathological neurology and neurochemistry, as well as the impact of multiple physiological systems [5,6]. The investigation of the potential relationship is made even more important by the global prevalence of both COVID-19 and neurodegenerative illnesses [7]. In this context, we present a case of sCJD manifesting at an unusual age, following a COVID-19 infection.

## Case presentation

An 83-year-old elderly man complained of fever, cough, decreased appetite, and loss of smell on April 15, 2021. In addition to standard blood tests, a SARS-CoV-2 polymerase chain reaction (PCR) test was performed because of the ongoing pandemic. The diagnosis of COVID-19 infection was confirmed by the elevated erythrocyte sedimentation rate (ESR), C-reactive protein, positive PCR, and lung computed tomography (CT) scan, which revealed bilateral multiple focal and patchy ground glass opacities with reticulation that were primarily subpleural. He was advised to follow the recommended home treatment plan, which showed noticeable improvement within two weeks. The patient had a past medical history of diabetes mellitus, hypertension, and right tibial angioplasty performed in 2018.

One year later, on August 18, 2022, he was brought by his son to the hospital with complaints of memory impairment, mood instability, being unable to use a home glucometer, urinary incontinence, and refusing feeds for three months. He was afebrile; his blood pressure was 160/100 mm Hg; his heart rate was 99/min; his respiratory rate was 18/min; and his oxygen saturation in room air was 92%. On examination, there was a clear memory impairment as the patient was unable to identify or name his son, and generalized mild muscle rigidity was noted. There was no evidence of nuchal rigidity or other meningeal signs. Examinations of the abdomen and chest turned up nothing unusual.

Given the symptoms, the possibility of a neurological issue was considered. This issue could have multiple etiologies, including inflammatory, neurovascular, toxic-metabolic, malignant, and autoimmune.

**Figure 1:**
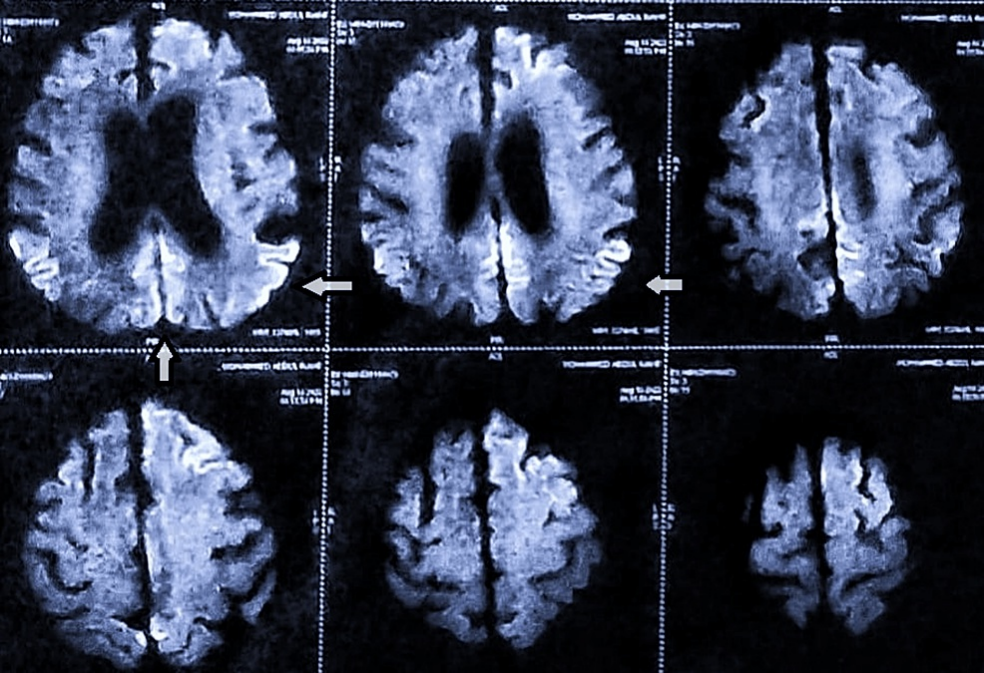
Diffusion-weighted MRI showing diffuse restriction of diffusion in the bilateral frontotemporoparietal regions.

**Figure 2:**
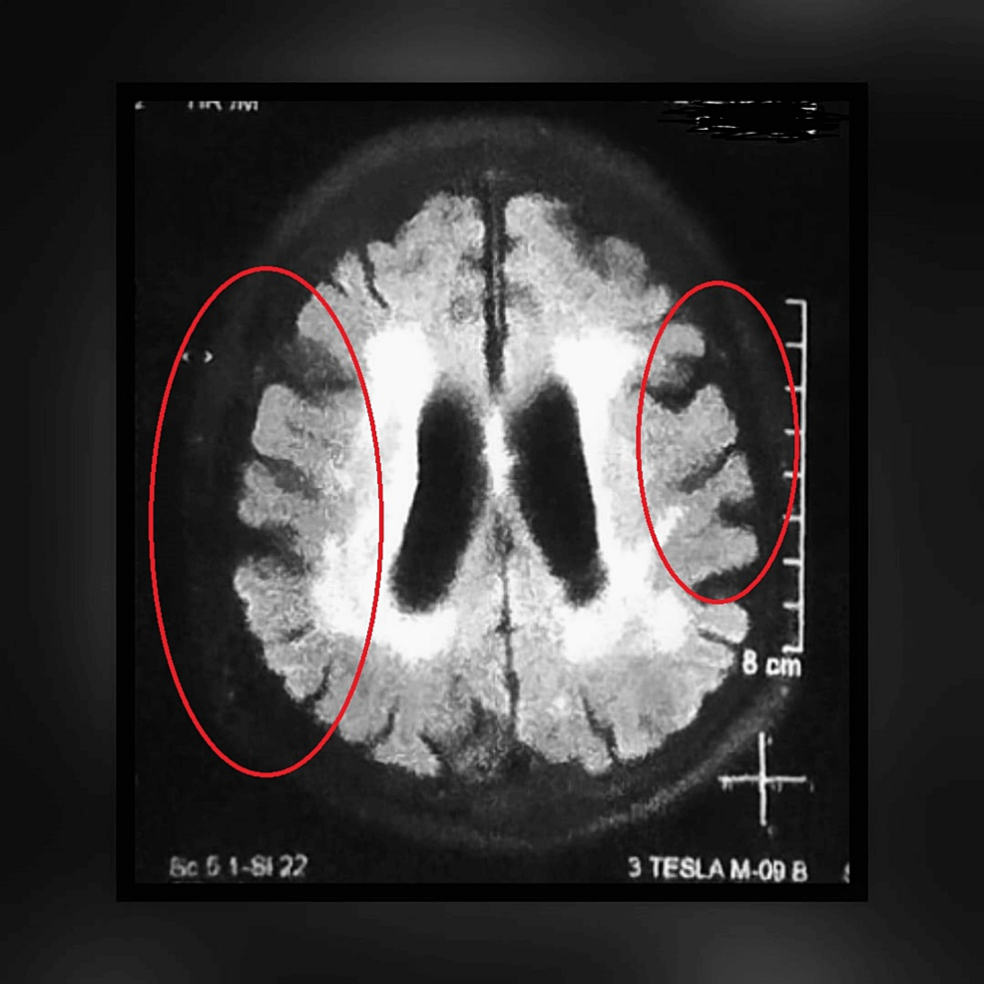
Magnetic resonance imaging (MRI) of brain showing cortical ribboning typical of Creutzfeldt Jakob disease.

All the baseline laboratory investigations were performed. Tests for blood biochemistry, serum electrolytes, and thyroid, kidney, and liver function were all normal. The results of the metabolic panel screening, serum vitamin B12, and folate levels, as well as viral markers (hepatitis B surface antigen, hepatitis C antibodies, and HIV 1 and 2 antigen and antibody), were all negative (Table 1). His HbA1c and plasma glucose levels were elevated as the patient has been non-compliant with his medications. An ultrasound of the abdomen and pelvis was done to rule out any malignancy. Magnetic resonance imaging (MRI) of the brain and MR angiography showed diffuse restriction of diffusion in the cortex of the bilateral frontotemporoparietal region (Figure 1) and cortical ribboning (Figure 2), suggesting a diagnostic possibility of encephalitis, CJD, or hypoglycemic encephalopathy. Hypoxic-ischemic injury is considered less likely based on the imaging characteristics observed. Additionally, evidence of senile cerebral atrophy accompanied by small vessel ischemia was noted, indicating possible underlying vascular pathology contributing to the observed MRI findings.

**Table 1:** Laboratory results. BUN: blood urea nitrogen, SGPT: serum glutamate pyruvate transaminase, SGLT: serum glutamate oxaloacetic transaminase, GGT: gamma-glutamyl transaminase, WBC: white blood cells, ESR: erythrocyte sedimentation rate, CRP: C-reactive protein.

Investigation	Result	Reference
Renal function tests		
BUN	14.9 mg/dl	4.67–23.4
Creatinine	0.8 mg/dl	0.8–1.3
Uric acid	5.70 mg/dl	3.5–7.2
Urea	32 mg/dl	17–43
Liver function tests		
Bilirubin-direct	0.21 mg/dl	0–0.2 mg/dL
SGPT	12 U/L	<50
SGLT	72 U/L	<50
GGT	32 U/L	<55
Complete blood picture		
Hemoglobin	13.19 g/dL	13–17
WBC	5700 cells/cu.mm	4000–10,000
Platelets	415,100/cumm	150,000–450,000
Vitamin B12	342 pmol/L	138–652
Folate	16.6 nmol/L	7.0–46.4
ESR	9 mm/hr	<15 mm/hr
CRP	6.95 mg/L	0–5.0
Viral markers		
Hepatitis B antibody	>500 mIU/mL	
HIV 1 and 2 Ab	Non-reactive	
COVID-19 PCR	Negative	

**Figure 3:**
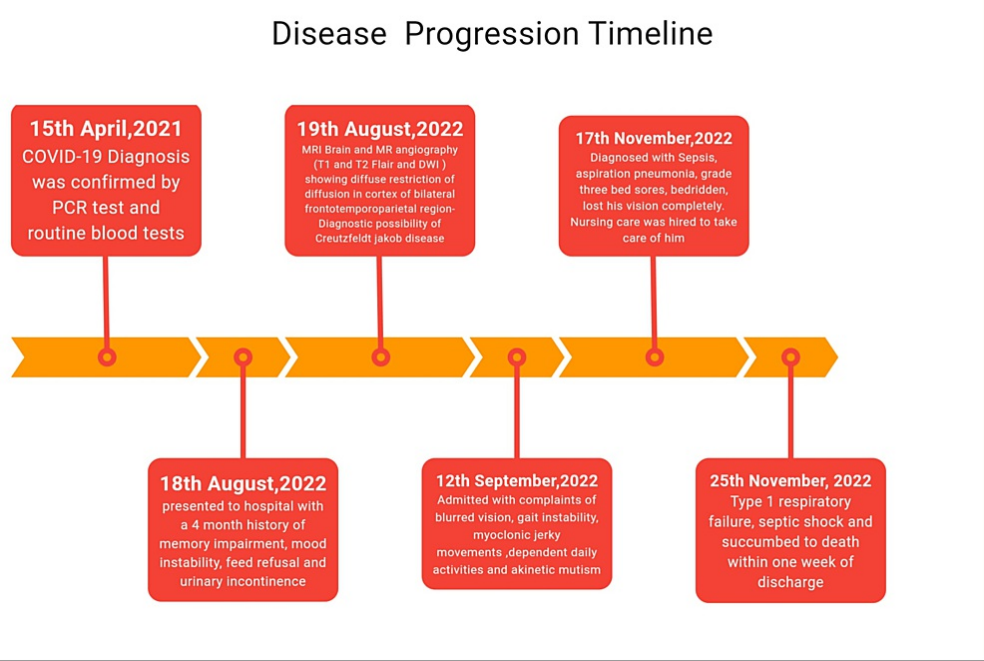
Disease progression timeline.

The patient's rapidly progressing dementia, the results of the examination and laboratory findings, and the MRI ruled out the possibility of any infectious, neurovascular, or hypoglycemic cause. The patient was advised to be admitted for further testing and confirmation, but refused. In less than a month, his condition began to worsen, and he was brought to the hospital with complaints of blurred vision, sporadic high fevers, gait instability, jerky movements of his head and left upper and lower limbs, and difficulty performing daily activities such as dressing or using the restroom. A neurological examination showed poor, higher mental status with a GCS of 10/15. Upon evaluation, he was diagnosed with sepsis, aspiration pneumonia, and grade 3 bed sores, for which he was managed appropriately. His rapidly progressing dementia, myoclonic jerks, visual and cerebellar disturbances, akinetic mutism, and an MRI confirmed a probable diagnosis of CJD with complications (Figure 3). The rapid progression of the disease necessitated the start of Ryle's tube feeding.

Regrettably, within a week of discharge, the patient became bedridden and lost all verbal communication. He had completely lost his vision by then. Nursing Care was hired to take care of him. When he was readmitted in November 2022, the patient had already entered into a state of type 1 respiratory failure and septic shock. He was treated with antibiotics, antiemetics, antiepileptics, and other supportive care management and was discharged with stable vitals; he was drowsy but not arousable, responded to deep stimuli, and was advised homecare. Sadly, the patient died within one week of discharge.

## Discussion

The patient was diagnosed with CJD because of the presentation of rapid progression of neuropsychological symptoms like cognitive impairment, myoclonus, and mutism, in addition to brain MRI findings. According to the Centers for Disease Control (CDC), probable CJD can be defined as having rapidly progressing dementia with at least two of the following: myoclonus, cerebellar or visual symptoms, signs of a pyramidal or extrapyramidal tract, and akinetic mutism and favorable results on a minimum of one of the subsequent lab assessments: positive 14-3-3 CSF analysis, atypical electroencephalography, and MRI imaging findings [8]. Our patient’s sCJD symptoms started 14 months after the COVID-19 infection, demonstrating a causal relationship. A few previous cases have reported similar outcomes [7,9,10]. Infections, heart disease, and respiratory illnesses claim the lives of over 80% of patients within the first year [11], a trend that is also evident in our patients.

Out of all the neurodegenerative diseases, CJD is the rarest. It typically manifests in individuals between the ages of 50 and 70, and it affects both genders equally [12]. The presentation of CJD outside the common age range is possibly seen in our case, and it is important not to rule out CJD if the patient shows features similar to the disease.

As our presented case illustrates, brain MRI may suggest the initial diagnosis of CJD, so radiological evaluation is an essential part of the initial workup for nonspecific neurodegenerative disorders [13]. The frontal cortex has been added to the list of five cortical regions of interest, in tandem with the temporal, parietal, precuneus, and occipital, corresponding to the recently proposed criteria by Bizzi et al. [14]. sCJD was suggested by rapidly progressing dementia, myoclonus, extrapyramidal signs, visual and cerebellar symptoms, akinetic mutism, hyperintense signal on diffusion-weighted imaging (DWI), and fluid-attenuated inversion recovery (FLAIR) sequences involving the temporal, parietal, and frontal cortex, as well as senile cerebral atrophy [8,15].

Since India is a developing nation, few cases have been reported. A total of 30 patients were documented between 1971 and 1990, with 20 cases of CJD confirmed and 10 cases suspected. In another case that Rasheed et al. reported, 10 individuals were identified as probable cases of sCJD during the 2011-2013 time frame [16]. Our case would be the first to be reported after so many years.

Overall, sCJD is difficult to diagnose as it can present in a variety of ways. Our case also demonstrates the difficulties encountered in diagnosing sCJD, as the patient presented with psychiatric disturbances that mask the neurological findings. Healthcare providers and psychiatrists must be diligent in the identification of CJD since many patients with sCJD exhibit psychiatric symptoms at the beginning of the course of the condition [17].

## Conclusions

This case emphasizes the importance of raising awareness of the variety of neurological symptoms linked to COVID-19, including uncommon illnesses like Creutzfeldt-Jakob disease and the unusual occurrence of disease outside the common age. It also highlights the need for heightened awareness of the diverse neurological manifestations associated with COVID-19, including rare conditions such as Creutzfeldt-Jakob disease. Clinicians should maintain a high index of suspicion for neurological complications in COVID-19 survivors, particularly in patients with atypical presentations or delayed onset of symptoms. Early recognition and appropriate management are crucial for improving outcomes in these challenging cases.
